# Physical and Chemical Processes and the Morphofunctional Characteristics of Human Erythrocytes in Hyperglycaemia

**DOI:** 10.3389/fphys.2017.00606

**Published:** 2017-08-30

**Authors:** Victor V. Revin, Natalia A. Klenova, Natalia V. Gromova, Igor P. Grunyushkin, Ilia N. Solomadin, Alexander Y. Tychkov, Anastasia A. Pestryakova, Anna V. Sadykhova, Elvira S. Revina, Ksenia V. Prosnikova, Jean-Christophe Bourdon, Nikolai Zhelev

**Affiliations:** ^1^Department of Biotechnology, Bioengineering and Biochemistry, Faculty of Biotechnology and Biology, Federal State-Financed Academic Institution of Higher Education, National Research Ogarev Mordovia State University Saransk, Russia; ^2^Department of Biochemistry, Biotechnology and Bioengineering, Faculty of Biology, Federal State-Funded Educational Institution of Higher Professional Education, Samara State University Samara, Russia; ^3^Division of Cancer Research, Ninewells Hospital and Medical School, University of Dundee Dundee, United Kingdom; ^4^CMCBR, Abertay University Dundee, United Kingdom

**Keywords:** human erythrocytes, hyperglycaemia, hemoglobin system, membrane lipids, proteolytic activity

## Abstract

**Background:** This study examines the effect of graduated hyperglycaemia on the state and oxygen-binding ability of hemoglobin, the correlation of phospholipid fractions and their metabolites in the membrane, the activity of proteolytic enzymes and the morphofunctional state of erythrocytes.

**Methods:** Conformational changes in the molecule of hemoglobin were determined by Raman spectroscopy. The structure of the erythrocytes was analyzed using laser interference microscopy (LIM). To determine the activity of NADN-methemoglobinreductase, we used the P.G. Board method. The degree of glycosylation of the erythrocyte membranes was determined using a method previously described by Felkoren et al. Lipid extraction was performed using the Bligh and Dyer method. Detection of the phospholipids was performed using V. E. Vaskovsky method.

**Results:** Conditions of hyperglycaemia are characterized by a low affinity of hemoglobin to oxygen, which is manifested as a parallel decrease in the content of hemoglobin oxyform and the growth of deoxyform, methemoglobin and membrane-bound hemoglobin. The degree of glycosylation of membrane proteins and hemoglobin is high. For example, in the case of hyperglycaemia, erythrocytic membranes reduce the content of all phospholipid fractions with a simultaneous increase in lysoforms, free fatty acids and the diacylglycerol (DAG). Step wise hyperglycaemia in incubation medium and human erythrocytes results in an increased content of peptide components and general trypsin-like activity in the cytosol, with a simultaneous decreased activity of μ-calpain and caspase 3.

**Conclusions:** Metabolic disorders and damage of cell membranes during hyperglycaemia cause an increase in the population of echinocytes and spherocytes. The resulting disorders are accompanied with a high probability of intravascular haemolysis.

## Introduction

Various physiological, pathological and nutritional conditions such as physical activity, large amounts of sweet food, emotional stress, metabolic syndrome, and diabetes are accompanied by high level of glucose in blood plasma. The high content of glucose in plasma accelerates the probability of non-enzymatic glycosylation of proteins, which induce damage to the cell membrane due to nonspecific aggregation of protein molecules and changes in protein-protein and protein-lipid interactions (Vasilyeva, 2005).

Taken together, these changes initiate the rapid aging of cells and the human organism. Metabolic syndrome significantly accelerates the development of atherosclerotic vascular damage and provokes earlier disability and death. During metabolic syndrome, which is currently the most common pathology of metabolic disorders, glycosylation of erythrocytic membrane proteins induces the impairment of rheological parameters of blood, low deformability and mobility of erythrocytes, high aggregation of erythrocytes and thrombocytes, high blood viscosity, and arterial hypertension (Shilov et al., 2008).

In addition, glycosylation of erythrocytic membrane proteins and hemoglobin during hyperglycaemia increases adhesion to endothelial cells, resulting in membrane destabilization (change in the asymmetry of membrane phospholipids), changes in viscoelastic properties of cells and their morphology (Riquelme et al., 2005). Taken together, these changes can impair the oxygen-transport function of erythrocytes and reduce erythrocyte lifespan. Furthermore, the number of damaged circulating cells and, aging erythrocytes will increase (Lang et al., 2006; Mindukshev et al., 2010).

The biochemical mechanisms of impaired growth in human erythrocytes during the development of hyperglycaemia have not been sufficiently investigated. In particular, there are scarce data on the composition and status of the lipid phase of the membranes, the relationship of these processes with the activity of methemoglobin formation and the activity of apoptotic enzymes. Moreover, there is a lack of data in the literature on the effect of these processes on the morphofunctional state of erythrocytes and their oxygen-transport properties.

Therefore, we aimed to perform a comprehensive study of the effects of graduated hyperglycaemia on the composition of phospholipids, the activity of proteolytic enzymes, and, the consequent effect of ongoing processes on the morphofunctional state of erythrocytes and the oxygen-transport properties of hemoglobin.

## Materials and methods

### Blood samples

This study was performed using pure erythrocyte fractions isolated from freshly obtained, anticoagulated donor blood from the regional station of blood transfusion. The average age of the donors was 28.4 ± 1.7 years. The research was approved by the Local Ethics Board at Mordovia State University in accordance with the principles of Good Clinical Practice (protocol number 12 of 17 September 2014). Informed consent statements were signed by all donors participating in the experiment.

Erythrocytes were washed 4 times with PBS until pure fractions were formed and centrifuged at 600 g for 10 min at temperature +4°C. Incubation of the erythrocytes was performed in a Ringer's solution at a ratio of 2:1 at 37°C for 30 min with gentle mixing every 5 min (to avoid haemolysis). For incubation, a Ringer's solution was used containing as follow (in mM/l): NaCl–136.75; KCl–to 2.68; NaHCO_3_–11.9 per; Na_2_HPO_4_–0.342; MgCl_2_–0.105; CaCl_2_–1.8, glucose–5 (normoglycaemia).

### Hyperglycemia model

Hyperglycaemic conditions were characterized by glucose content, in which the graduated concentration was accompanied by an equimolar decrease in the content of sodium chloride: 10 mM/l glucose–NaCl: 131.75 mM/l; 15 mM/l glucose–NaCl: 126.75 mm/l; 20 mM/l NaCl; 121.75 mM/L. The incubation medium was separated by centrifugation in the same manner, and erythrocytes were analyzed using spectrophotometry (Zavodnik and Lapshina, 1996) to determine the correlation of the hemoglobin forms.

### Conformation and properties of hemoglobin

Conformational changes in the hemoglobin molecule were determined by Raman scattering on In Via Renishaw (UK) microscope with a short-focus high-luminosity monochromator (focus distance–250 mm).

For excitation of the Raman spectra we used a laser with a wavelength of 532 nm, max radiation power of 100 mW, and 100 × objective. The data recorder was a –CCD detector (1,024 × 256 pixels with Peltier cooling to −70°C) with a grating of 1800 lines/mm. The digitized spectra were processed in WIRE 3.3 (part of the unit's software). To analyse the conformation of globin haematoporphyrin, we used specific bands of the RS spectrum, which enabled an estimate of the relative amount of oxyhemoglobin and the ability of hemoglobin to bind to and isolate ligands, including oxygen (Brazhe et al., 2009).

### Laser interference microscopy

The structure of the erythrocytes was analyzed using laser interference microscopy (LIM) *in vitro* (Byazhe et al., 2006; Yusipovich et al., 2011; Revin et al., 2016) using MII-4 sysytem (Russia). The measurements were performed at room temperature, and a suspension of erythrocytes in the incubation medium (1:2) was placed on mirror glass. The smear was prepared and covered with cover glass. Images of 10 sites, with a monolayer arrangement of cells in an interference channel, were obtained, with reflected light in each sample. The images were processed using FIJI (Schindelin et al., 2012). The structure of the erythrocytes was assessed by registering the average value of the optical path difference (OPD) and phase image area using at least 100 cells from each sample. The phase volume of the erythrocyte was calculated using the following formula:

$$\begin{array}{l} {\text{V}}_{\text{cell}}={\text{F}}_{\text{mean}}\cdot {\text{S}/\text{n}}_{\text{cell}}-{\text{n}}_{\text{m}} \end{array}$$

Where F_mean_ is the mean value of the optical path difference, proportional to the thickness of erythrocyte; S is the phase image area of the cells; n_*cell*_ is the refractive index of erythrocyte, equal to 1.405; n_m_ is the refractive index of the surrounding solution (1.333).

### Determination of the activity of NADN-methemoglobinreductase

To determine the activity of NADN-methemoglobinreductase, we used the P.G. Board method (Board, 1981). The degree of glycosylation of the erythrocyte membranes was determined using a method previously described by Felkoren et al. (1991). Before defining the proteolytic enzymes, the suspension of erythrocytes was haemolysed by the addition of a buffer of 20 mM three-HCl containing 2 mM EDTA, pH 7.5 in a ratio of 1:9. The haemolysate was maintained at a temperature 2-4°C for 15 min and centrifuged at 16,000 × g for 40 min.

The supernatant was used to determine the activity of μ-calpain with the release of enzyme using ion exchange chromatography (column 3 × 15; DEAE-cellulose) and eluted by a gradient of 0.1–0.4 M NaCl. Next, the mean calpain activity of the fractions was determined using incubation medium previously described by Sorimachi et al. (1997) (imidasole buffer, 4% casein, 50 mM of CaCl_2_, 50 mM of cysteine) (Stroev et al., 1991; Elce John, 2000; Sorimachi et al., 2000). Then, the general proteolytic activity, in ascending order of the absorption level at a wavelength of 280 nm, was measured following incubation of the haemolysate and protein deposition by 5% trichloroacetic acid (TCA) (Bazarnova et al., 2008).

The release of fractions with calpain activity occurred during movement in a reverse gradient from 0.2 to 0.1 M of sodium chloride immediately after the release of hemoglobin. The activity of μ-calpain was calculated as the difference between activity with and without the inhibitor in the incubation medium (phenylmetylsulfonylfluoride, 2 mM PMSF, Sigma, USA). The content of peptides in the incubation medium and erythrocytes was determined using the Lowry method, with the Bio-Rad DC Protein Assay a set of protein assay Dc reagents (Bio-Rad). The content of peptides in the erythrocytes was determined after deproteinisation by 5% of TCA. The active concentration of caspase-3 in erythrocytes was recorded using an enzyme immunoassay (BD Biosciences, USA) with Stat Fax 3200 microplate reader (USA).

### Analysis of membrane phospholipids

To analyse the state of the membrane phospholipids, we isolated membranes from the haemolysate using 5 mM NaH_2_PO_4_+ 0.5 mM PMSF (phenylmetylsulfonylfluoride) solution, cooled to 0°C, pH 8.0 in a ratio of 1:20. The mixture was incubated for 10 min at 4°C and then centrifuged at 20,000 × g for 40 min (0°C). The supernatant was removed and the residue was resuspended in lysis solution and centrifuged in the same manner. The sample was washed three times. Lipid extraction was performed using the Bligh and Dyer method (Bligh and Dyer, 1959).

To separate the phospholipid fractions we used one-dimensional chromatography combined with chloroform/methanol/glacial acetic acid/water in a ratio of 60/50/1/4 (Evans et al., 1990). Chromatographic separation was performed in a thin layer of silica gel deposited on a glass plate. Standard plates from HPTLC Silicagel 60 F254 (Merck. Germany) were used. To separate DAG and FFA we used the combination of heptane/diethyl ether/glacial acetic acid (60/40/2 by volume). Detection of the phospholipids was performed using V. E. Vaskovsky method (Vaskovsky et al., 1975).

The amount of phospholipid fractions and free fatty acids in the erythrocytic membranes was determined using a densitometer TCL Scanner 3 (Gamag, Switzerland) at an absorption wavelength of 360 nm using a deuterium lamp and winCATC software.

### Statistical analysis

The statistical processing was performed using the Statistika 0.06 software package. To determine the significance levels, the Student's test was used. Repeats in the variation series of different indicators were from 8 to 20 values.

## Results

Hyperglycaemic conditions are characterized by a low affinity of hemoglobin to oxygen, which is manifested as a parallel decrease in the content of hemoglobin oxyform and the growth of desoxyform (Table 1).

**Table 1:** Correlation of hemoglobin formation (%), content of glycosylated hemoglobin (g/l) and the degree of glycosylation of membrane proteins (rel. units).

**Index**	**Degree of hyperglycemia, mM/l**			
	**5**	**10**	**15**	**20**
DesoxyHb, %	39.320 ± 0.337	39.927 ± 0.295	40.841 ± 0.321^*^Δ	40.470 ± 0.123^*^
OxyHb, %	58.359 ± 0.230	57.676 ± 0.356^*^	56.280 ± 0.286^*^Δ	56.779 ± 0.251^*^
MetHb, %	2.321 ± 0.022	2.397 ± 0.023^*^	2.899 ± 0.042^*^Δ	2.751 ± 0.033^*^
Membrane-boundHb, %	8.172 ± 0.259	10.430 ± 0.448^*^	12.100 ± 0.994^*^Δ	15.888 ± 0.427^*^Δ
GlycatedHb, Γ/π	6.220 ± 0.221	6.667 ± 0.448	7.083 ± 0.083	10.833 ± 0.397^*^Δ
Degree of glycosylation of membrane proteins, rel.units.	0.109 ± 0.002	0.182 ± 0.007^*^	0.206 ± 0.008^*^	0.304 ± 0.019^*^

* P < 0.05 as related to normoglycemia;

Δ *P < 0.05 as related to previous degree of hyperglycemia*.

Upon a 30-min incubation of the cells, an increase in the glycosylation of hemoglobin occurred only when the glucose concentration was 20 mM/l, whereas a small but reliable growth in methemoglobin formation, increase in membrane-bound form of hemoglobin and degree of glycosylation of proteins in erythrocytic membranes has been previously observed when the glucose level exceeded a two-fold level (10 mm/l) (Table 1).

The data obtained using the spectrometric measurements were confirmed by Raman scattering (Table 2).

**Table 2:** Analysis of RS spectra of human erythrocytes hemoglobin in hyperglycaemia.

**Exposure**	**Relative amount of oxyhemoglo-bin I_1375_/ (I_1355_+I_1375_)**	**Relative ability of hemoglobin to bind ligands including oxygen I_1355_/I_1550_**	**Relative ability of hemoglobin to isolate ligands I_1375_/I_1580_**	**Hb complex with nitric oxide during destruction of the link between protein and hemoporphyrin, regulates the ability of Hb to release O_2_ I_1668_/I_1580_**	**The intensity of symmetric and asymmetric oscillations of the pyrrole rings (related to conformational changes of pyrroles I_1375_/I_1172_**	**Affinity of hemoglobin to ligands, to oxygen in the first place (I_1355_/I_1550_/ (I_1375_/I_1580_)**
	**M ± m**	**M ± m**	**M ± m**	**M ± m**	**M ± m**	**M ± m**
5 mM/l of glucose	0.493 ± 0.003	0.625 ± 0.012	0.479 ± 0.009	0.131 ± 0.010	1.579 ± 0.060	1.309 ± 0.019
10 mM/l of glucose	0.483 ± 0.001^*^	0.718 ± 0.023^*^	0.517 ± 0.011	0.142 ± 0.004	1.248 ± 0.026^*^	1.385 ± 0.024
15 mM/l of glucose	0.481 ± 0.001^*^	0.652 ± 0.013^*^	0.485 ± 0.009	0.141 ± 0.005	1.179 ± 0.026^*^	1.345 ± 0.014
20 mM/l of glucose	0.482 ± 0.002^*^	0.675 ± 0.007^*^	0.522 ± 0.006	0.120 ± 0.007	1.293 ± 0.026^*^	1.295 ± 0.011Δ

* P < 0.01 as related to normoglycemia;

Δ *P < 0.01 as related to previous degree (level) of hyperglycemia*.

We found a reliable decrease in the relative content of oxygenated hemoglobin, with an average of 2%. Moreover, we obtained similar spectrophotometric percentages of hemoglobin forms (Tables 1, 2). The conformational changes observed indicate an increased ability of hemoglobin to bind to ligands and the reliable occurrence of low hemoglobin affinity to oxygen in the case of 20-mM/l hyperglycaemia, at approximately 11%. Furthermore, there is a low intensity of symmetric and asymmetric vibrations of pyrrole rings, which indicated a less conformational mobility of the haeme structures and an impairment of the effective binding of ligands (Table 2).

At 15- and 20-mM/l glucose hyperglycaemia, we detected a high level of lysophosphatidylcholine (LFH) in the erythrocyte membrane, and the content of phosphatidylcholine (PFS) was reliably low under in conditions of increasing amounts of glucose, i.e., up to 10 mM/l. In addition, we recorded a low level of sphingomyelin (SM) and fractions of phosphatidylinositol + phosphatidylserine (PI + PS). Strong hyperglycaemia (20 mm/l) is characterized by a low content of all fractions with a simultaneous increase of in LPC (Table 3).

**Table 3:** Phospholipid composition of erythrocytic membranes under normoglycaemia and graduated hyperglycaemia.

**Phospholipid**	**N◦ of peak**	**Rf**	**5**	**10**	**15**	**20**
			**mkgP/mg of lipid**			
LPC	1	0.13	0.058 ± 0.003	0.023 ± 0.001	0.078 ± 0.004^*^	0.093 ± 0.005^*^
SM	2	0.21	0.808 ± 0.040	0.471 ± 0.024^*^	0.678 ± 0.034^*^	0.348 ± 0.017^*^
PC	3	0.34	0.346 ± 0.017	0.147 ± 0.007^*^	0.252 ± 0.013^*^°	0.107 ± 0.005^*^°
PI+PS	4	0.58	1.239 ± 0.062	0.632 ± 0.032	1.017 ± 0.051^*^	0.527 ± 0.026^*^
PEA	5	0.9	0.768 ± 0.038	0.359 ± 0.018^*^	0.608 ± 0.03°	0.185 ± 0.009^*^
PC/PEA			0.45	0.41	0.41	0.59

* P < 0.01 as related to normoglycemia;

° *P < 0.01 as related to previous level*.

The percentage of increased lysophosphatides under strong 20-mm/l hyperglycaemia was 160%. The SM level was 2 to 3 times lower, with PC–3 times; PI+PS–2.35%; and PEA–4 times. The low content of phospholipids and high content of lysophosphatides indicated the activation of phospholipase A and C, which was accompanied by loosening of the membrane and higher ion permeability (Mills and Needham, 2005).

Analysis of the content of free fatty acids (FFA) and the product of the reaction catalyzed by phospholipase C and diacylglycerol (DAG) showed growth that was proportional to the level of hyperglycaemia, and the percentage of growth with 20 mM/l hyperglycaemia is 133 and 182%, respectively (Table 4).

**Table 4:** Content of free fatty acids (FFA) and DAG, in mcg FA/mg of total lipids.

**Index**	**Degree of hyperglycemia**			
	**5 mM/l (norm)**	**10 mm/l**	**15 mM/l**	**20 mM/l**
FFA	15.0 ± 0.7	28.0 ± 0.9^*^	31.0 ± 1.3^*^	35.0 ± 1.5^*^°
DAG	11.0 ± 0.6	17.0 ± 0.8^*^	20.0 ± 1.1^*^	31.0 ± 1.4^*^°

* P < 0.01 as related to normoglycemia;

° *P < 0.01 as related to the level of 10 mM/l*.

These results clearly demonstrate the activity of phospholipase.

The normal correlation of phospholipid fractions pre-determines the effective regulation of active and passive transport of substances, the sensitivity of cells to the action of ligands, and the activity of membrane-bound enzymatic systems. The optimal operation of ion-carrying systems (Ca^2+^ and Na^+^, K^+^-ATP-PS) is facilitated by stable PEA content. During severe hyperglycaemia (20 mm/l), a sharp decrease in PEA content will be accompanied by a defect in ion-carrying processes (Table 3). Moreover, it will trigger a general change in the structure of the membrane phospholipid bilayer, as PEA is a structural phospholipid (Delaunay, 2002).

Importantly, the low content of PEA causes a disorder in endoglobular homeostasis and inhibition of the antioxidant activity of cells in (Afanasiev et al., 2007). The low content of phospholipids with polyunsaturated fatty acids (PC, PI, PEA) triggers defatting of membranes and a high correlation of cholesterol/PL, which is accompanied by changes in physico-chemical properties, namely, high microviscosity. These findings indicate that hyperglycaemia is followed by a disruption in membrane permeability.

In hyperglycaemia, human erythrocytes demonstrate a high content of peptide components, which was specifically high when the content of glucose in the incubation medium was 10 mm/l (Table 5). A further increase in the level (degree) of hyperglycaemia was accompanied by an increased release of protein compounds into the incubation medium due to higher cell membrane permeability (Table 5).

**Table 5:** Content of protein and peptide compounds in the incubation medium and peptides in erythrocytes (during normo- and hyperglycaemia).

**Indicators**	**Content of glucose in incubation media. mM/l**			
	**5**	**10**	**15**	**20**
Content of proteins and peptides in incubation media. μm/ml	71.6 ± 4.8	84.3 ± 5.3	114.9 ± 5.7^*^	101.7 ± 5.1^*^
Content of peptides in erythrocyte. μg/ml	106.5 ± 6.6	189.9 ± 6.3^*^	160.9 ± 5.9^*^	125.4 ± 6.2°

* P < 0.01 as related to normoglycemia;

° *P < 0.05 as related to normoglycemia*.

Measurement of the proteolytic activity revealed interesting patterns. The release of μ-calpain fractions and identification of the average activity of the enzyme showed a decrease with a parallel increase in the degree of hyperglycaemia, which was statistically significant when the concentration of glucose reached 20 mM/l. A low activity of caspase 3 was detected, which was also strongest under conditions of severe hyperglycaemia (Table 6). Increased proteolytic activity can be detected only by the identification of the overall trypsin-like activity of cytosol with respect to the high content of peptides in the haemolysate, without the isolation of enzymes and addition of third proteolytic substrates (Table 6).

**Table 6:** Activity of erythrocyte enzymes during graduated hyperglycaemia.

**Enzyme activity**	**Concentration of glucose in incubation media, mM/l**			
	**5**	**10**	**15**	**20**
NADH-methemoglobinreductase, μM/min · gHb	19.545 ± 3.801	14.760 ± 3.878^*^	27.858 ± 2.446^*^	20.685 ± 5.019
μ-Calpain, μM/Min · mg of protein	15.414 ± 2.317	11.979 ± 1.398	11.593 ± 1.489	9.130 ± 1.402^*^
Caspase-3 activity, ng/ml	0.562 ± 0.029	0.443 ± 0.035^*^	0.401 ± 0.027^*^	0.345 ± 0.025^*^°
Trypsin-like activity, membrane fraction, μg/ml·h	0.159 ± 0.079	0.135 ± 0.075	0.151 ± 0.072	0.171 ± 0.079
Trypsin-like activity, cytosol, μg/ml·h	2.15 ± 0.025	2.70 ± 0.015^*^	3.29 ± 0.041^*^°	3.48 ± 0.030^*^°

* P < 0.01 as related to normoglycemia;

° *P < 0.05 as related to normoglycemia*.

**Figure 1:**
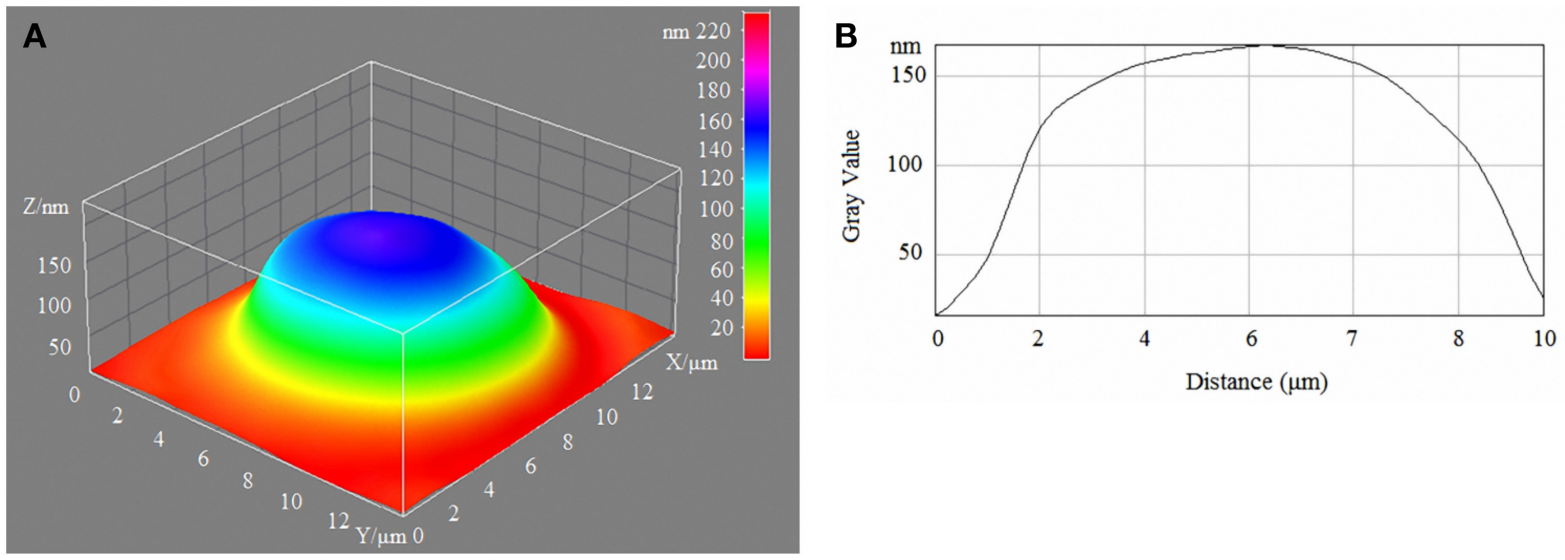
3D model **(A)** and profile **(B)** of human erythrocytes in normoglycaemia.

**Figure 2:**
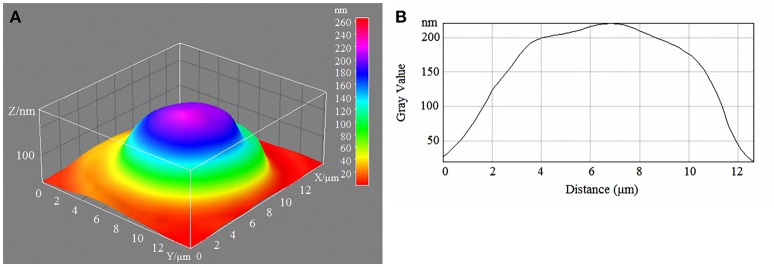
3D model **(A)** and profile **(B)** of human erythrocytes incubated in 10-mM/l glucose media.

**Figure 3:**
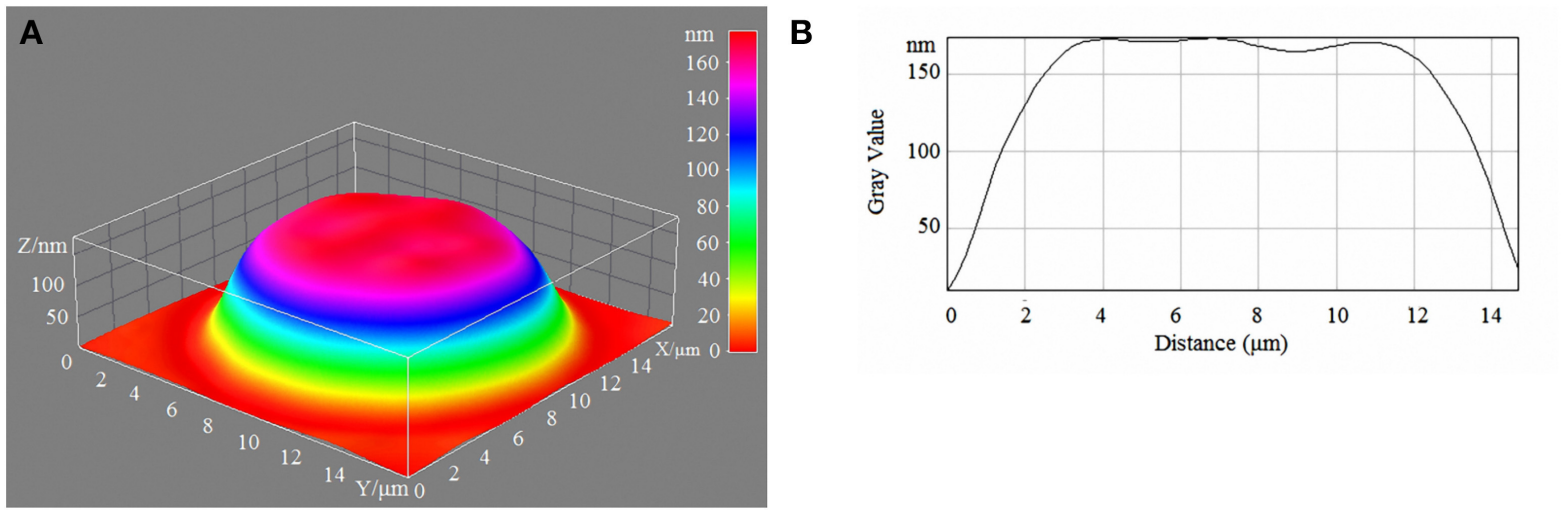
3D model **(A)** and profile of human erythrocytes **(B)** incubated in 15-mM/l glucose media.

**Figure 4:**
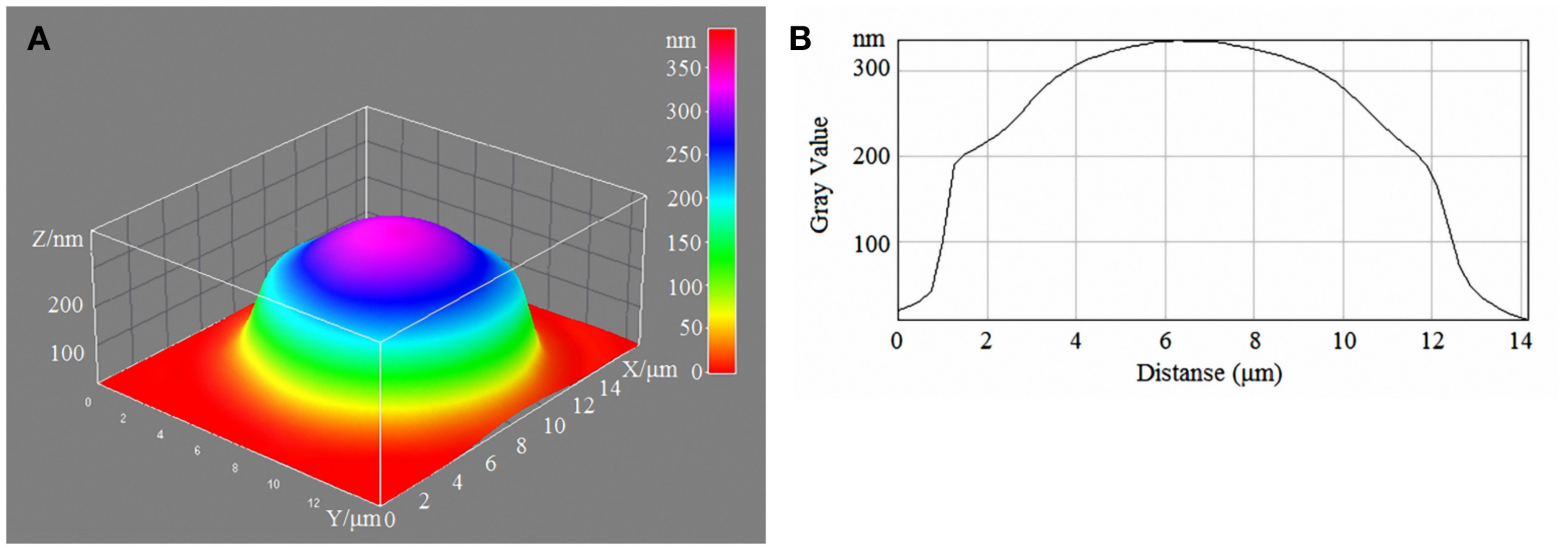
3D model **(A)** and profile of human erythrocytes **(B)** incubated in 20-mM/l glucose media.

Laser interference microscopy showed distinct structural changes of erythrocytes with increased concentrations of glucose in the incubation medium (Table 7, Figures 1–4).

**Table 7:** Morphological parameters of erythrocytes in normo- and hyperglycaemia, according to LIM results.

	**Phase area of erythrocyte, S, μm^2^**	**Mean phase height, Φ_mean_, nm**	**Phase volume of erythrocyte, V, μm^3^**	**Physical (geometrical) width of erythrocyte, Z_mean_, μm**	**Hemoglobin weight in erythrocyte, mHb, pg**
5 mM/l of glucose	43.92 ± 1.42	125.54 ± 1.45	73.60 ± 2.78	1.67 ± 0.19	52.66 ± 1.99
					26.28 ± 0.99
10 mM/l of glucose	45.44 ± 0.78	161.11 ± 1.49^*^	97.46 ± 2.08^*^	2.14 ± 0.20	34.93 ± 0.74
15 mM/l of glucose	78.91 ± 2.42^*^	147.16 ± 4.50	154.82 ± 5.14^*^	1.96 ± 0.18	55.20 ± 1.84^*^
20 mM/l of glucose	59.16 ± 1.31^*^	245.03 ± 3.56^*^Δ	193.26 ± 5.23^*^Δ	3.26 ± 0.05^*^	69.42 ± 1.87^*^

* P < 0.01 as related to normoglycemia;

Δ *P < 0.01 as related to previous level of hyperglycemia*.

During normoglycaemia, erythrocytes appeared similar to discocytes, with a normal distribution of hemoglobin inside the cells. During hyperglycaemia, the estimated parameters were high overall. In addition to the relative areas of the erythrocytes, all indicators were high in conditions of 20-mm/l hyperglycaemia (Table 7).

With a high content of glucose in the incubation medium, echinocytes accumulated in the population of cells, and the cell profile demonstrated visible outgrowths on the erythrocyte surface. At up to 15-mm/l glucose, spherocytes appeared, and the cells appeared more swollen (Figure 3). At 20-mm/l glucose, the cells were slightly compressed compared with 15-mm/l glucose; however, other changes became more robust (Table 7, Figure 4).

## Discussion

Disorder in the metabolic and functional state of human erythrocyte membranes at a high concentration of glucose in the incubation medium occurs primarily due to the general activation of biochemical and physicochemical processes in cells. The shift in the dissociation curve in the direction of deoxygenation causes increased methemoglobin formation as DOHb, which is less resistant to auto-oxidation compared tooxyform (Zavodnik and Lapshina, 1996; Ivanov, 2001). The gradual reduction of the conformational mobility of haeme structures (Table 2) is most likely caused by the high degree of the glycosylated protein component of hemoglobin, its permeability in the membrane, followed by a gradual increase in membrane-bound hemoglobin (Table 1).

The observed increase in the degree of glycosylation of membrane proteins induces changes in the state of membrane phospholipids. The change in protein-lipid interactions results in the activation of membrane phospholipase, causing an increase in LPC and DAG, and results in a high content of FFA in erythrocytic membranes. Membrane permeability under these conditions increases, and the peptide and protein compounds are released into the incubation medium. Some reduction in their release at 20-mm/l hyperglycaemia may be accounted for by the low activity of μ-calpain and caspase 3 due to less affinity to the glycosylated form of hemoglobin. However, their content in the erythrocytes and incubation medium was higher compared to normoglycaemia, most likely due to the continued high trypsin-like activity of the cell cytosol.

Metabolic disorders and injury to the cell membrane trigger morphological changes in erythrocytes. Previous research findings have shown a gradual accumulation of structurally damaged cells in the population. First, in 10-mM/l hyperglycaemia, the number of echinocytes increases (reversible morphological changes) and the phase volume and thickness of the cells increases. Next, stomacytes and spherocytes appear in the population. LIM microscopic results indicated strong cell swelling. In 20-mm/l hyperglycaemia, the number of spherocytes increased. Furthermore, the cells showed some shrinkage, but the ratio of surface/volume values reached a critical level. Thus, haemoglytic destruction is very likely.

The destruction of erythrocytes in the bloodstream in hyperglycaemia will result in a chronic inflammation response, which can damage the body's blood vessels.

Changes in the erythrocytic membrane not only result in morphological disorders of the whole erythrocyte, but they can also consequently result in conformational changes in hemoglobin as well as its oxygen-binding and oxygen-transport function. The low content of oxyhemoglobin, weak symmetric and asymmetric oscillations of pyrrole rings and increase in hemoglobin affinity to ligands, including oxygen, has been demonstrated.

## Conclusion

Taken together, we demonstrate the significant risk of the prolonged availability of high glucose concentrations in plasma. Nonspecific glycosylation of membrane proteins and erythrocytesin hemoglobin results in a weak affinity of hemoglobin to oxygen and its loss by cells enroute to tissues in the human body. Moreover, the resulting damage to membranes and cell metabolism increase the probability of an accumulation of functionally defective aging erythrocytes in the circulating population. More rapid spherization of erythrocytes, in the absence of physiological mechanisms of apoptosis, introduces the possibility of necrotic cell death in the bloodstream, resulting in the gradual development of chronic states of hypoxia and inflammation.

## Author contributions

VR: designed experiments, interpreted data, wrote manuscript. NK, NG, IG, IS, AT, AP, AS, and ER: designed and performed experiments wrote manuscript. KP: performed experiments and analyzed results. NZ and JCB: made substantial contribution to the analysis and interpretation of the data, revised and critically reviewed the manuscript.

### Conflict of interest statement

The authors declare that the research was conducted in the absence of any commercial or financial relationships that could be construed as a potential conflict of interest.
